# Risk factors and prevention of liver cancer: A bibliometric and visual analysis

**DOI:** 10.1097/MD.0000000000035740

**Published:** 2023-11-24

**Authors:** Min Yang, Huiqin Zhang, Jieqiu Zhang, Xiaopeng Yao

**Affiliations:** a School of Public Health, Southwest Medical University, Luzhou, China; b School of Medical Information and Engineering, Southwest Medical University, Luzhou, China; c Central Nervous System Drug Key Laboratory of Sichuan Province, Southwest Medical University, Luzhou, China.

**Keywords:** Bibliometrics, liver cancer, prevention, risk factors, visualization

## Abstract

Liver cancer has become an important public health problem. In this study, bibliometrics and visual analysis were performed on the literature related to the risk factors and prevention of liver cancer, in order to understand the latest research progress of the risk factors and prevention of liver cancer. The Web of Science database was used as a retrieval platform to retrieve the published research results from 2012 to 2023. CiteSpace and VOSviewer were utilized for bibliometrics and visual analysis. A total of 2388 articles were screened according to exclusion criteria. Between 2012 and 2018, the number of articles published fluctuated. From 2018 to 2023, the number of published documents showed a steady upward trend. The 3 journals with the most publications are World Journal of Gastroenterology, PLOS ONE, and Hepatology. The United States and China are the countries with the most publications, while Harvard University, the National Institutes of Health and the University of Texas System are the 3 institutions with the most publications. Keywords such as hepatitis B virus, hepatitis C virus, alcohol, obesity, recrudescence rate, global burden are hot words in the field of liver cancer risk factors and prevention. The current research mainly focuses on the influence of environmental factors, behavioral lifestyle and biological factors on liver cancer, as well as the primary and secondary prevention of liver cancer, but there are still many undetermined factors to be explored.

## 1. Introduction

Liver cancer is the fourth most common cause of cancer-related death worldwide^[1–3]^ and can be divided into primary and secondary categories.^[4,5]^ The etiology and exact molecular mechanism of primary liver cancer (PLC) are not fully understood. This review mainly discusses the risk factors and prevention of PLC. Liver cancer was the second leading cause of cancer life lost globally between 2005 and 2015, with absolute years of life lost increasing by 4.6% (95%CI −1.6%–15.4%), placing a heavy burden of disease on families and society.^[6,7]^ Based on global cancer statistics, Rumgay et al predicted^[8]^ that between 2020 and 2040, the incidence of liver cancer will increase by 55.0% and the number of deaths will increase by 56.4%. It is estimated that by 2025, the incidence of liver cancer worldwide will exceed 1 million cases.^[9]^ We can see that PLC has been and will continue to be a major threat to global health.^[10]^ Therefore, a comprehensive understanding of the risk factors for liver cancer is very important for screening high-risk individuals and preventing liver cancer.^[11]^ The main known risk factors for hepatocellular carcinoma (HCC) are chronic hepatitis B infection, chronic hepatitis C infection, alcohol abuse, nonalcoholic fatty liver disease and the effects of aflatoxin.^[12–15]^ Smoking and diabetes are suspected risk factors.^[16–19]^ In addition, there are some dietary factors^[20,21]^ that are protective factors for liver cancer, but are not understood by the public, thus hindering the adoption of preventive measures. However, some behavioral and lifestyle factors^[22,23]^ are mistaken by the public as risk factors for liver cancer, but in fact, there is no exact evidence for the occurrence of liver cancer, which leads people to take wrong preventive measures. Therefore, it is necessary to conduct a comprehensive review of the latest risk factors for liver cancer.

Bibliometrics is a method of quantitative literature analysis using mathematics, statistics and other sciences to explore the quantity, distribution, rule and relationship of the literature to be studied, which can comprehensively reflect the knowledge base and development trend of various research fields.^[24,25]^ Although there have been reviews on the analysis of independent risk factors for liver cancer, there are few comprehensive systematic reviews on the risk factors and prevention of liver cancer at present, and few studies use bibliometric methods to comprehensively analyze this field.

To study the current status and trends in the field of risk factors and prevention of liver cancer, this review selected relevant literature from 2012 to 2023. This review analyzed the article characteristics, author data, keywords and hotspots in the field of risk factors and prevention of PLC with the help of bibliometric, and identified the information of high publication journals, high publication authors, current hotspots and future trends in this field, which can provide reference for related researchers.

## 2. Methods

### 2.1. Data sources and search strategies

**Figure 1.**
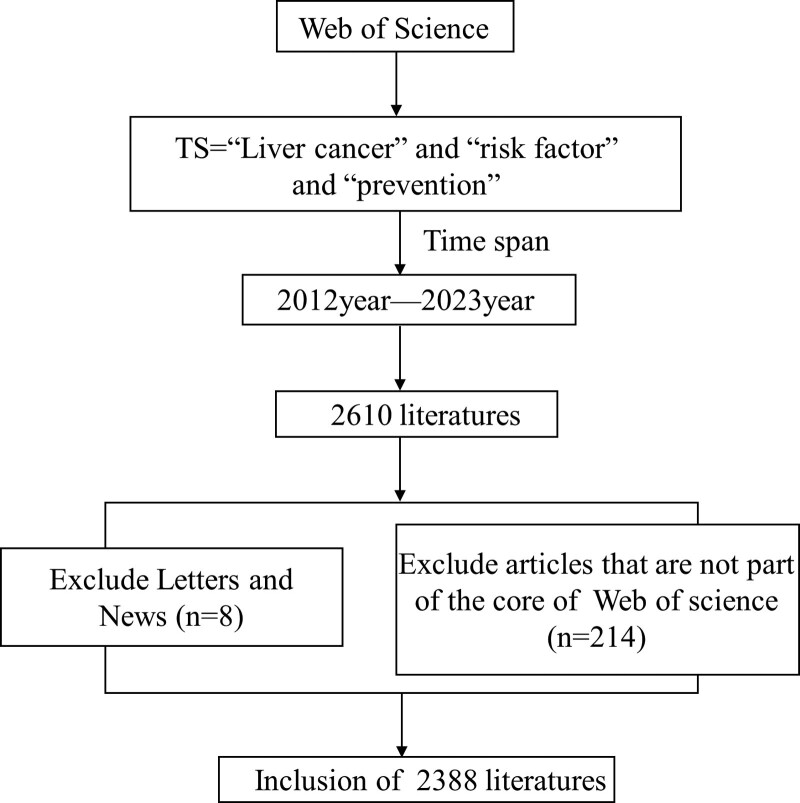
Diagram of paper search and screening process.

The WoS Core Collection platform was used to search the literature related to the risk factors and prevention of liver cancer from 2012 to 2023. The search terms were used as follows: (ALL = (Liver cancer) AND ALL = (risk factor) AND ALL = (prevention)) and the time period spanned from 2012 to 2023. Inclusion criteria: literature topics related to liver cancer risk factors and prevention, the literature was published between January 1, 2012 and July 1, 2023. Exclusion criteria: non-article types such as letters and news. Literature not in the Web of science core database. A total of 2388 papers were finally obtained (Fig. 1).

### 2.2. Bibliometric and visual analysis

To describe all literature on liver cancer risk factors and prevention, Web of Science (http://wcs.webofknowledge.com) was used to analyze retrieval results. We converted all data that met the requirements from the WoS Core Collection to Text File format and imported the data into CiteSpace V6.2.R4 analysis software. Parameter setting: Set Time Slicing to January 2012 to July 2023 and Years Per Slice to 1 year. We select “Country,” “Author,” and “keyword” as node types respectively. We set the selection criteria as q-index (k = 25) and TOP N = 50, and the “Pruning sliced networks” in “Pathfinder” were sliced as Pruning. CiteSpace was used to perform the bibliometric analysis. Visualization knowledge maps consist of nodes and links. Different nodes in a map represent elements such as a cited reference, institution, author, and country, and links between nodes represent relationships of collaboration or cooccurrence.

A network visualization map based on data searched from the Web of Science Core Collection database was created using VOSviewer (www.vosviewer.com) to analyze all keywords, it is helpful to summarize the research hotspot of liver cancer risk factors and prevention.

## 3. Result

### 3.1. Comprehensive analysis of published papers

#### 3.1.1. Quantity and trends analysis of published papers.

**Figure 2.**
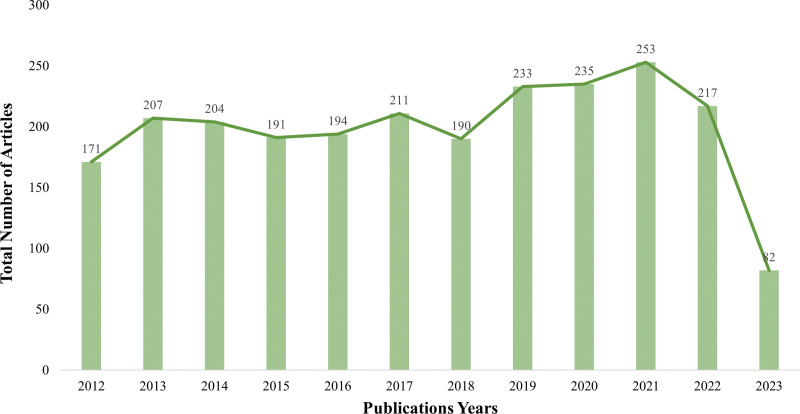
Trend chart of the number of published articles from 2012 to 2023.

A total of 2388 articles were included. From 2012 to 2021, the overall number of published papers (Fig. 2) showed an increasing trend. The number of articles published after 2022 has decreased slightly. The trend can be divided into 2 stages. From 2012 to 2018, the number of published articles fluctuated. In 2013, the number of published articles increased the fastest and reached the highest, reaching 207. From 2018 to 2021, the number of published documents showed a steady rising trend.

#### 3.1.2. Analysis of journals.

The top 10 journals with the most published articles are analyzed. Table 1 shows the names of the top 10 journals as well as the number of articles, Influence factor, and other information. The total number of published articles accounts for more than 25% of the total number of articles in this field. The top 3 journals by number of articles were World Journal of Gastroenterology (5.9%), PLOS ONE (5.6%) and Hepatology (2.6%).

**Table 1:** Risk factors and prevention of liver cancer journal rankings (N = 2388).

Rank	Journal or proceedings	Publications	Percentage (n/N)	JCI	IF (2022)	JCR	Country
1	World Journal of Gastroenterology	65	2.7	0.82	4.3	Q2	China
2	PLOS ONE	59	2.5	0.91	3.7	Q2	United States
3	Hepatology	50	2.1	3.01	13.5	Q1	United States
4	Journal of Hepatology	41	1.7	4.96	25.7	Q1	Netherlands
5	Nutrients	40	1.7	1.04	5.9	Q1	Switzerland
6	Cancer Epidemiology Biomarkers Prevention	39	1.6	0.94	3.8	Q2	United States
7	Gastroenterology	39	1.6	5.37	29.4	Q1	United States
8	Cancers	33	1.4	0.92	5.2	Q2	Switzerland
9	Journal of Gastroenterology and Hepatology	30	1.3	0.8	4.1	Q2	Australia
10	International Journal of Cancer	29	1.2	1.31	6.4	Q1	Switzerland

IF = Impact factor, JCI = Journal Citation Indicator, JCR = Journal Citation Reports.

#### 3.1.3. Analysis of countries.

**Figure 3.**
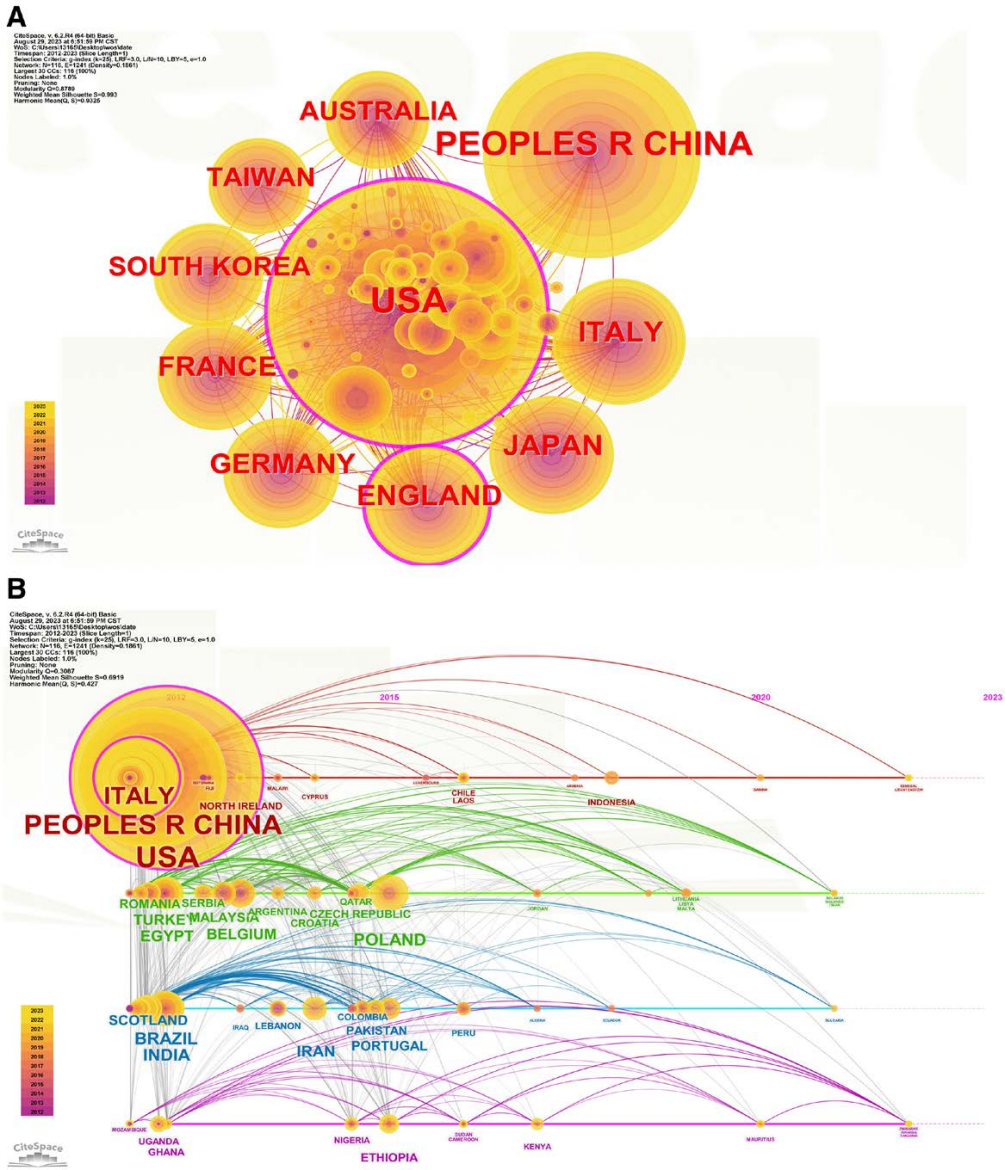
Network visualization map for top publication countries/regions. (A) CiteSpace was used to establish the top 10 countries and common clusters. (B) CiteSpace was used to analyze the clustering timelines of major countries in these 12 yr.

This review analyzes the top 10 countries/regions with the highest number of publications, as shown in Figure 3A: USA, PEOPLES R CHINA, ITALY, JAPAN, ENGLAND, GERMANY, FRANCE, SOUTH KOREA, TAIWAN and AUSTRALIA. We analyzed the clustering timelines of major countries in the past 12 years through CiteSpace. In the earliest timelines, we could find that some Central Asian countries, such as China and India, were the main ones. These countries have dense timelines and more cooperation links. The details are shown in Figure 3B.

#### 3.1.4. Analysis of organizations.

The study analyzed the top 10 institutions with the highest number of publications. The institution here mainly refers to the primary institution to which the author belongs. Table 2 shows that the top 3 institutions are Harvard University (4.8%), National Institutes of Health (NIH, USA) (3.8%), and UDICE French Research Universities (3.4%).

**Table 2:** Ranking of institutions to which the first authors belonged (N = 2388).

Rank	Institution name	Publications	Percentage (n/N)
1	Harvard University	100	4.2
2	National Institutes of Health NIH, USA	91	3.8
3	UDICE French Research Universities	82	3.4
4	NIH National Cancer Institute NCI	75	3.1
5	University of California System	75	3.1
6	Institute National De La Sante Et De La Recherche Medicale Inserm	70	2.9
7	University of Texas System	66	2.8
8	Harvard Medical School	65	2.7
9	International Agency for Research on Cancer	54	2.3
10	Harvard T.H.Chan School of Public Health	49	2.1

NCI = National Cancer Institute, NIH = National Institutes of Health, UDICE = French Research Universities.

### 3.2. Comprehensive analysis of author

#### 3.2.1. Main researchers.

This study analyzes the first authors of articles in this field over the last 12 years. The top 10 first authors are ranked according to the number of published papers, which are detailed in Table 3. Specifically, Jemal, Ahmedin, Freedman, Neal David and McGlynn, Katherine A are listed as the top 3 authors with the most published articles.

**Table 3:** Ranked by the number of articles published by the author (N = 2388).

Rank	Authors	Publications	Percentage (n/N)
1	Jemal, Ahmedin	24	1.0
2	Freedman, Neal David	22	0.9
3	McGlynn, Katherine A.	22	0.9
4	Simon, Tracey G.	17	0.7
5	Jenab, Mehdi	15	0.6
6	Chung, Raymond	14	0.6
7	La Vecchia, Carlo	13	0.5
8	Chan, Andrew T.	13	0.5
9	Siegel, Rebecca L.	13	0.5
10	Zhang, Xuehong	12	0.5

#### 3.2.2. Analysis of co-authorship networks.

**Figure 4.**
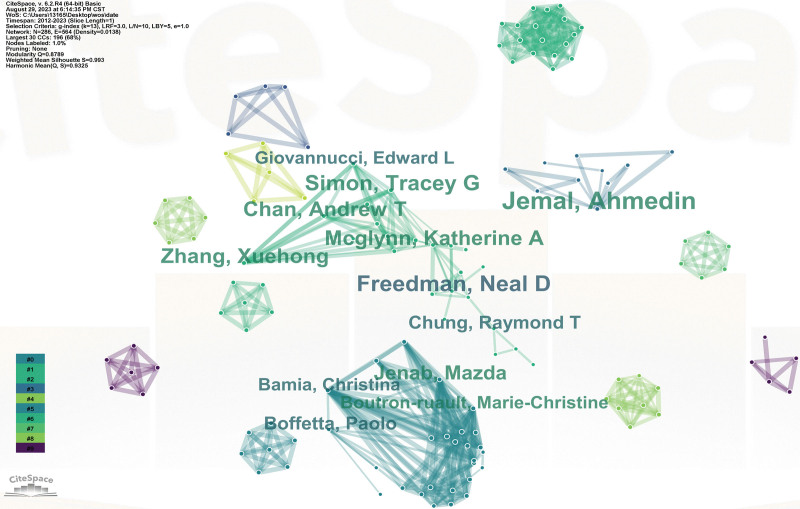
Author cluster co-occurrence analysis. Different colors represent different clusters, and the main clusters are labeled with the author name.

In this study, CiteSpace was used to analyze the state of collaboration among authors. Figure 4 shows the cluster of top authors in this field and the distribution of collaboration time. The author cluster can be divided into 9 main clusters according to the time represented by the line colors. There are 3 core clusters represented by Jemal, Ahmedin, Freedman, Neal David and McGlynn, Katherine A, as well as some small scattered clusters.

### 3.3. Analysis of research trend and burst detection with keywords

**Figure 5.**
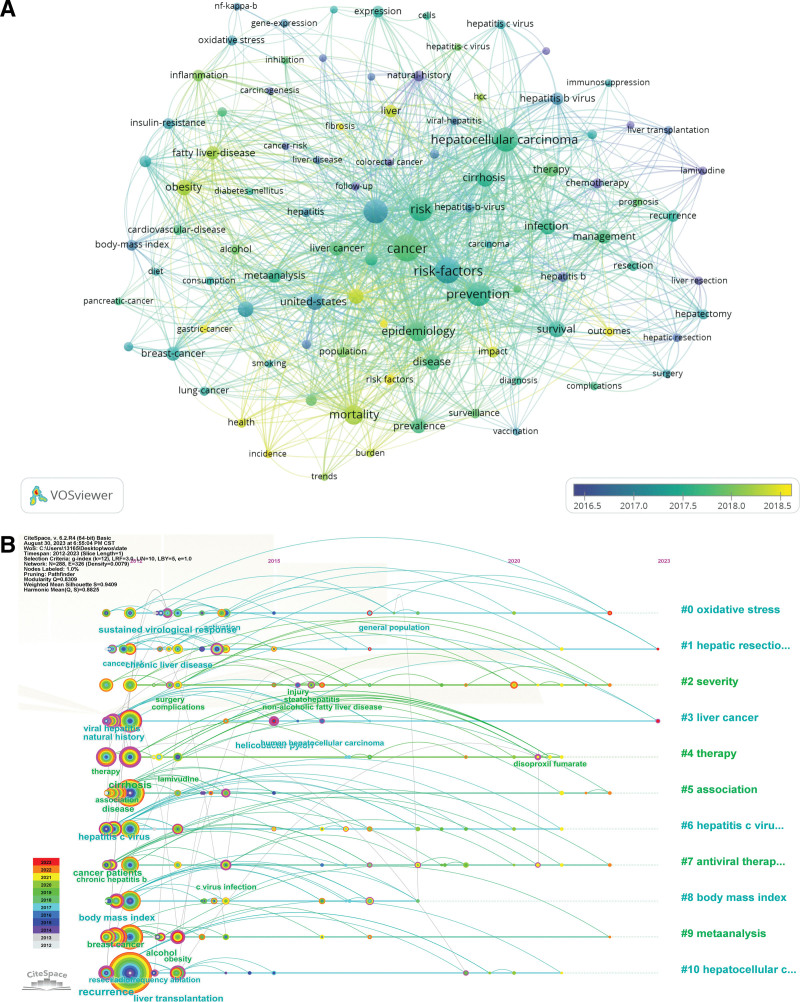
The keywords and hot topics during 2012 to 2023 were analyzed visually. (A) Co-occurrence analysis of the most popular keywords over the decade was performed using VOSviewer. (B) The time line analysis of hot words in the past 12 yr is carried out by CiteSpace.

**Figure 6.**
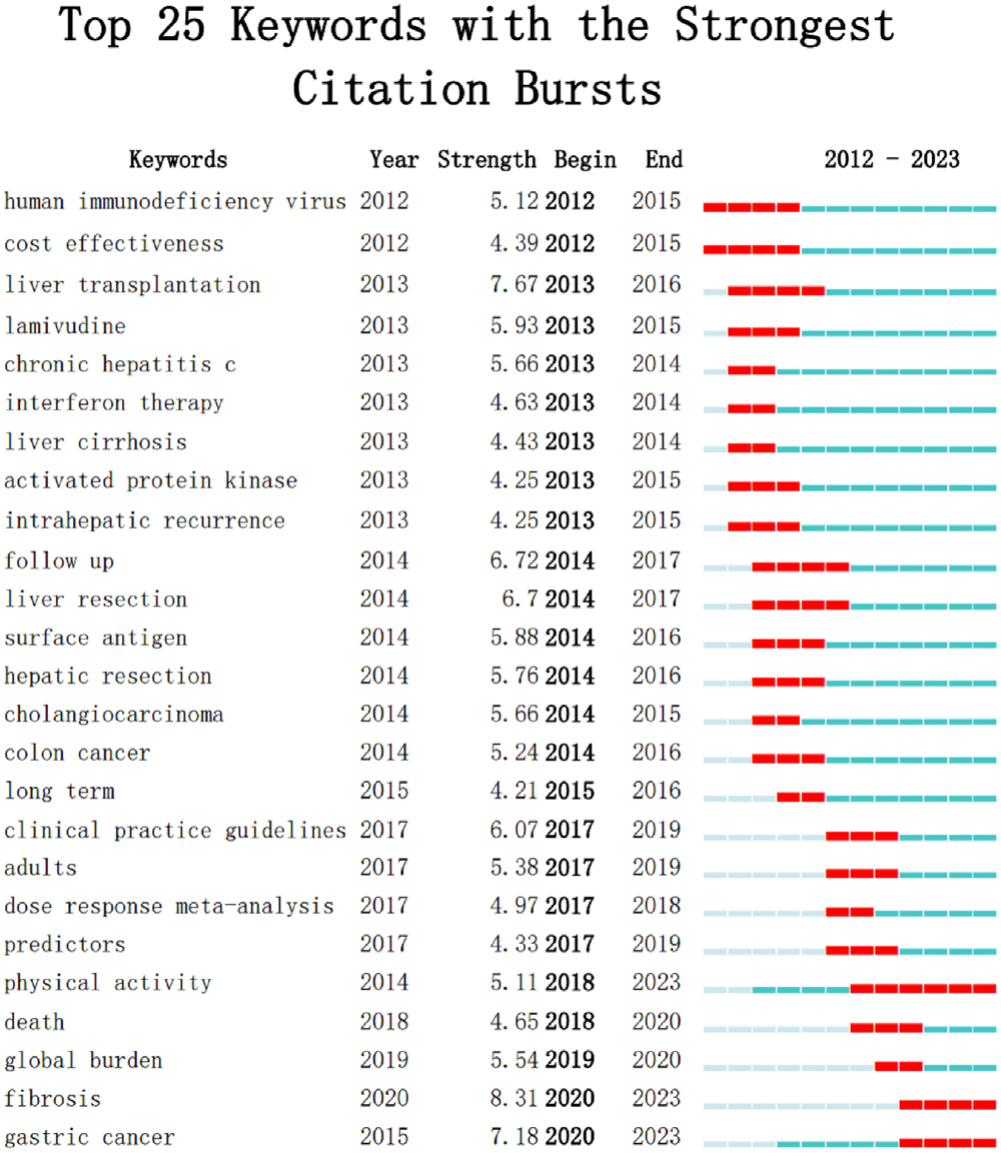
From 2012 to 2023, 25 keywords with high frequency emerged. Visual analysis of the outbreak intensity of 25 hot words with the highest outbreak rate in the last 12 yr.

Through keyword analysis, the research hotspots and trends can be indirectly revealed. In this study, VOSviewer and CiteSpace are used for keyword visualization (Fig. 5A), keyword timeline analysis (Fig. 5B) and visual analysis of the outbreak intensity of 25 hot words with the highest outbreak rate in the last 12 years (Fig. 6). The study found that key words such as hepatitis B virus (HBV), hepatitis C virus (HCV), alcohol, obesity, recrudescence rate, global burden are the risk factors of liver cancer and the prevention literature mentioned more hot words. In general, physical activity is the hot spot with high outbreak degree and the longest research time span. From 2014 to 2023, it has been the hot spot of research, with the highest outbreak intensity reaching 5.11.

## 4. Discussion

In this study, we conducted bibliometrics and visual analysis of liver cancer risk factors and prevention articles published in the Web of Science database over the past decade. In order to better understand the research in this field, we conducted a detailed analysis of the research in the field of liver cancer risk factors and prevention from 2012 to 2023 to determine the current status and trends of the research.

### 4.1. Characteristics of papers in liver cancer risk factors and prevention

Based on the trend of the number of published papers in this field, the development of this field in the past decade can be divided into 2 stages. From 2012 to 2018, this stage was a slow development stage, and the number of articles in this field fluctuated. However, 2014 was the year with the largest number of publications and the fastest growth rate in this period. Since 2014, safe and effective oral antiviral drugs have been available for HCV,^[26]^ and the prevention of HCV can reduce the occurrence of HCC. Therefore, many scholars may have increased their interest in this field in 2014, resulting in the fastest increase in the number of published papers in this year. In the other stage, from 2019 to 2021, the number of publications in this stage increases year by year, which is a stage of rapid development and popularity. The death rate of liver cancer rose from the third place in 2018 to the second place in 2020. The epidemiological burden of liver cancer is constantly increasing,^[27]^ coupled with the general awareness of disease prevention in recent years, as well as the integration of public health field and clinical field, this field has developed rapidly in this stage.

The journal data are analyzed in detail by bibliometrics. In terms of Journal impact factor ranking, there are top medical journals such as Gastroenterology, Hepatology and Journal of Hepatology. Relevant articles in these journals represent the development frontier of this field. If you want to have a deep understanding of this field, these journals may be preferred. In summary, the top 10 journals with the most publications in this field are excellent in terms of journal impact factor, and other aspects. In addition to specialized liver disease journals, we can see some comprehensive journals or interdisciplinary journals, such as International Journal of Cancer, Cancer Epidemiology Biomarkers Prevention, etc. These journals are suitable for scholars in the clinical medical community or public health field to publish relevant research.

Most countries have participated in the research in this field, the United States, China, Italy, Japan and other countries are the most productive countries in this field. From the timeline graph (Fig. 3B), it can be noticed that Central Asian countries such as China and India, as well as some European countries, have produced uninterrupted results during the decade from 2012 to 2021. These countries have a high incidence of liver cancer and a serious disease burden.^[28]^ These countries have paid more attention to the field of liver cancer and made significant contributions to this field. Harvard University, NIH, USA, and UDICE French Research Universities are the 3 institutions that have published the most papers. Cooperation and exchanges between different countries and institutions can be strengthened to jointly promote the development of this field.

According to the author clustering analysis, 3 core clusters dominated by Jemal, Ahmedin, Freedman, Neal David and McGlynn, Katherine A are formed, and there are also some scattered small clusters. Among the top 10 authors in the number of published papers, McGlynn, Katherine A, Simon, Tracey G, Chan, Andrew T and other authors formed a major cluster, which was the cluster with the most connections to these 10 authors, and also the cluster with the longest connection line, including Freedman, Neal David and Chung, Raymond. Close collaboration among authors has contributed to a large body of literature in this area. Jemal Ahmedin has published the most papers in this field, mainly focusing on cancer epidemiology, cancer control and prevention. Freedman, Neal David, is the second most prolific author of the paper, which focuses on the influence of multiple risk factors on disease, such as chemical factors and physiological factors. McGlynn, Katherine A is the third most published scholar in this field, who mainly studies the epidemiology and disease burden of liver cancer, especially the exploration of risk factors of HCC and the risk of liver cancer. It can be seen that scholars have different research focuses, but they are all studying the risk factors and prevention of liver cancer. Scholars from different research directions can strengthen cooperation and produce more fruitful results. At the same time, if we want to know the latest progress in this field, we can pay more attention to the academic achievements of these scholars.

### 4.2. Current frontiers and trends in liver cancer risk factors and prevention

Through keywords visualization and hotspot breakout analysis, we found that the risk factors of liver cancer mainly focused on environmental factors, behavior and lifestyle, biological factors. The research on the prevention of liver cancer mainly focuses on primary prevention and secondary prevention.

Some chemical factors in environmental factors are related to the occurrence of liver cancer. For example, occupational exposure to vinyl chloride is associated with angiosarcoma,^[29]^ and microcystin exposure^[30,31]^ is also potentially associated with liver disease. Organochlorine exposure is a risk factor for liver tumors and HCC.^[32]^ Pesticides can also have an effect on cancer.^[33]^ A study in the United States showed that compared with non-farmers, farmers had a higher risk of HCC.^[16]^ Air pollutants and light pollution have potential effects on health and are positively correlated to induce some liver diseases,^[34–37]^ but whether they are risk factors for liver cancer can be further explored in the future.

Bad behavior and lifestyle, such as bad dietary habits, have an impact on the incidence of liver cancer. Alcohol and tobacco are proven carcinogens of liver cancer, and HCC is usually found in patients with alcoholic liver disease in the middle and late stages.^[38,39]^ Obesity caused by unreasonable diet can lead to an increased risk of nonalcoholic fatty liver disease and HCC,^[40]^ and the risk of liver cancer in obese people is increased by 87%.^[41]^ One study found a nonlinear association between high levels of fasting blood glucose and an increased risk of PLC in Chinese men.^[42]^

Biological factors are one of the causes of human tumors. Long-term infection and high viral load of HBV and HCV are dose-responsive to the risk of liver cancer.^[43]^ We need to pay attention to genetic factors. Maternal obesity has an impact on the tumorigenesis of their offspring.^[44]^ Studies have reported that individuals with CC gene type of MIR17HG polymorphism rs7318578 have an increased risk of liver cancer.^[45]^ The role of blood group in the pathogenesis of HCC remains controversial, and whether it is a risk factor for HCC remains to be discussed.^[46]^ Age and gender are risk factors for liver cancer, and the occurrence of HCC increases with age.^[47]^ A study stratified by age group showed that the risk of liver cancer in patients with liver disease was much higher than that in patients under 50 without liver disease.^[48]^ Compared with females, the incidence of liver cancer in males is 2.89 times.^[49]^

From the analysis results of keywords and hot topics, it can be found that in addition to the study of etiology, many hot topics such as health management, survival rate, follow-up, outcome also emerged. It can be seen that the current focus of attention on cancer is no longer solely clinical treatment, but also gradually shifted to focus on prevention, management and improvement of the quality of life of cancer patients, which also highlights the necessity of improving prevention strategies and implementing sustainable cancer control actions. Effective prevention of liver cancer has a great impact on reducing medical care expenditure and achieving good health benefits.^[50]^ The primary task of prevention is to find the risk factors associated with cancer risk,^[51]^ so exploring the risk factors of liver cancer is the key to implementing preventive measures.

Based on the above analysis of risk factors, the primary prevention of liver cancer is mainly through vaccination and changes in behavior and lifestyle. Firstly, vaccination is an effective primary prevention measure.^[52]^ The development of neonatal vaccination programs is conducive to reducing the incidence of chronic HBV infection in children and chronic HBV infection in young people in high-prevalence areas.^[53]^ Secondly, in terms of dietary intake, drinking coffee, tea, yogurt and intake of vegetables are beneficial to the prevention of liver cancer.^[54–58]^ At the same time, attention should be paid to the safe storage of food, the supply of clean water and the restriction of aflatoxin-contaminated food.^[59]^ For smoking, it is necessary to implement tobacco control, which can be carried out through non-drug intervention and drug intervention.^[60,61]^ Secondary prevention is also very important in the prevention of chronic diseases. Chronic HBV infection is usually asymptomatic and requires greater emphasis on screening.^[62,63]^ The government should raise the awareness of the public and doctors, accelerate the screening of high-risk groups,^[64,65]^ and implement effective policies and primary screening programs for liver cancer to achieve better health benefits.^[66,67]^ It is also important to avoid the negative effects of over-screening on disease. Prior to screening, further investigation is needed to determine the best surveillance strategy and screening target population to mitigate the risk of overdiagnosis.^[68]^

### 4.3. Limitations of the study

The study also has several limitations. The literature databases used were limited to Web of Science, so the literature database in this study is still not comprehensive. Secondly, English is still the preferred language for academic journals today, therefore we focused on papers published in English, which resulted in an omission of articles published in other languages.

## 5. Conclusion

Through bibliometrics and visualization software analysis, this study reveals the global research status and trend of liver cancer risk factors and prevention. Since 2017, the number of publications is basically increasing, and great progress has been made in the study of the etiology and prevention of liver cancer, a trend that shows the increasing attention of people. The United States and China have contributed more to this field, and Harvard University and the NIH, USA have published the most papers. Current research focuses on the impact of environmental factors, behavioral lifestyle and biological factors on liver cancer, as well as prevention and secondary prevention of liver cancer. Although great progress has been made in the investigation of the etiology and prevention of liver cancer in the past 12 years, there are still many undetermined factors, such as whether some air pollutants, light pollution, blood type and other factors are risk factors for liver cancer, as well as the improvement of liver cancer screening plans and prevention strategies still need to be explored. This requires extensive collaboration between different countries, institutions and academics, both domestically and internationally. By summarizing existing research hotspots and predicting future development trajectory, this study helps scholars in related fields to better grasp the development of etiology research and prevention of liver cancer, and provides references for them.

## Author contributions

**Conceptualization:** Min Yang, Xiaopeng Yao.

**Data curation:** Min Yang.

**Formal analysis:** Min Yang.

**Investigation:** Min Yang, Huiqing Zhang, Jieqiu Zhang, Xiaopeng Yao.

**Methodology:** Min Yang, Xiaopeng Yao.

**Project administration:** Min Yang, Xiaopeng Yao.

**Resources:** Xiaopeng Yao.

**Software:** Min Yang.

**Supervision:** Xiaopeng Yao.

**Validation:** Min Yang, Xiaopeng Yao.

**Visualization:** Min Yang, Xiaopeng Yao.

**Writing – original draft:** Min Yang.

**Writing – review & editing:** Huiqing Zhang, Jieqiu Zhang, Xiaopeng Yao.
